# Impact of Narrow Empiric Antibiotic Spectrum and Patient Characteristics on Clinical Outcomes in Bone and Joint Infections: A Retrospective Cohort Study

**DOI:** 10.3390/antibiotics15060620

**Published:** 2026-06-18

**Authors:** Lasse Bæk Krag, Anton Alexander Nolte Peterlin, Emil Gleipner-Andersen, Hans Gottlieb

**Affiliations:** 1 Department of Orthopedic Surgery, Horsens Hospital, 8700 Horsens, Denmark; 2 Department of Orthopedic Surgery, Herlev Hospital, 2730 Herlev, Denmarkhans.gottlieb.03@regionh.dk (H.G.); 3 Department of Veterinary and Animal Sciences, University of Copenhagen, 1870 Frederiksberg C, Denmark

**Keywords:** bone and joint infections, narrow spectrum, empiric, antibiotics, microbiological susceptibility, patient characteristics

## Abstract

**Background**: Bone and joint infections (BJIs) are a significant clinical challenge due to their tendency to recur, increased healthcare expenses, reduced quality of life, and mortality. Patients with BJIs are a heterogeneous group due to their different clinical presentations as well as patient-related risk factors. Empiric antibiotic regimens are commonly based on deductions from *in vitro* microbiologic findings, despite the fact that their relative efficacy and optimal antibiotic choices are underexplored. **Methods**: This retrospective cohort study included 521 patients surgically treated for BJIs at a specialized orthopedic infection unit between 2016 and 2023. Treatment strategies were guided by the Oral Versus Intravenous Antibiotics for Bone and Joint Infection (OVIVA) trial. All patients received a narrow-spectrum Gram-positive–targeted empiric systemic antibiotic regimen determined according to regional recommendations in collaboration with infectious disease specialists. The primary outcome was clinical failure within one year, with a minimum follow-up of 12 months. For the analyses, the patients were divided into three groups based on microbiological susceptibility: susceptible (SusEmp), non-susceptible (NonSus) and culture-negative (CulNeg) patients. **Results**: The three groups were found to differ significantly in seven patient-related factors: sex, age at primary operation (OP age), BMI, ASA group, diabetes status, peripheral arterial disease status (PAD), and endocrinopathy status (other than diabetes). In performing multivariate analyses, OP age was found to be independently associated with the overall failure rate (*p* = 0.04) and ASA group (*p* = 0.047), and PAD (*p* = 0.043) was independently associated with the secondary outcome of proximal amputation. Patients with non-susceptible pathogens (NonSus) had more than twice the odds of clinical failure (OR: 2.10; 95% CI: 1.12–3.95) and nearly fivefold higher odds of secondary proximal amputation (OR: 4.95; 95% CI: 1.41–23.2) compared with patients with susceptible pathogens (SusEmp). **Conclusions**: The current study demonstrates that a large group of patients can presumably be treated safely with a more restrictive narrow approach. More studies are needed to identify subgroups suited for the safe use of a narrow-spectrum empiric regimen, hereby reserving the broad-spectrum antibiotics for patients with the right indications and for whom it would have a positive effect on the clinical outcome. Such an approach would justify a more restrictive stewardship of broad-spectrum antibiotic use without negatively impacting patient outcomes.

## 1. Introduction

Bone and joint infections (BJIs) are a rising burden on the global healthcare system and remain a significant clinical challenge due to their tendency to recur, added healthcare expenses, reduced quality of life, and mortality [1]. BJI is a broad term, and its management is complicated by substantial heterogeneity not only in clinical presentation, ranging from implant-related infections such as fracture-related infections (FRIs) and prosthetic joint infections (PJIs) to diabetic foot osteomyelitis (DFO) and chronic osteomyelitis (cOM) [2], but also in patient-related factors, including demographics, comorbidity profiles, surgical history, immune status, and pathogen exposure [3]. This diversity challenges the construction of one-size-fits-all treatment strategies, which is further challenged by differences in local antimicrobial resistance patterns, diagnostic practices and available resources [4,5,6]. Clinical assumptions, including the unverified notion that systemically administered antibiotics reliably achieve therapeutic concentrations at the sites of infection in all patients, may also influence treatment regimens [7,8,9]. Despite these complexities, empirical systemic antibiotic regimens continue to be based on assumptions through deductions based on *in vitro* microbiologic results that neglect the complexity of all patient-related factors that impact the actual *in vivo* infection site [10]. While international consensus guidelines for the diagnosis of specific subtypes of BJI exist, such as FRIs [11] and PJIs [5], adherence to these guidelines remains variable, and empirical treatment strategies often reflect local microbiological profiles. As a result, the management of BJIs remains unstratified, complicating scientific comparisons and impeding the development of standardized treatment pathways [12]. Empiric broad-spectrum antibiotic regimens are commonly applied to ensure early pathogen coverage, pending culture results. However, the relative efficacy and optimal antibiotic choices are still under investigation [4,13,14,15].

A recent meta-analysis estimated that resistant infections incur additional costs ranging from $2371.4 to $29,289.1 per patient, with a mean excess hospital stay of 7.4 days (95% CI: 3.4–11.4) [16]. In 2021, bacterial antimicrobial resistance (AMR) was directly responsible for an estimated 1.14 million (95% uncertainty interval [UI]: 1.00–1.28) deaths, with an additional 4.71 million (95% UI: 4.23–5.19) deaths associated. Extrapolations for 2050 suggest AMR could lead to 1.91 million (95% UI: 1.56–2.26) attributable deaths and 8.22 million (95% UI: 6.85–9.65) associated deaths annually [17]. This global crisis underscores the need for antimicrobial stewardship and rational use, particularly in specialties like musculoskeletal infection, where empirical antimicrobials are common and bone penetration may be uncertain [8].

Despite varying approaches, clinical failure rates of 10–30% have consistently been reported among patients with BJIs [18,19,20]. The fact that a substantial proportion of tissue samples from BJI patients are culture-negative [21] further strengthens the need for empiric treatment to be based on clinical outcomes rather than deductions. Evidence evaluating the clinical importance of the empiric antimicrobial spectrum in surgically managed BJIs remains limited, and in the absence of outcome-based evidence, clinicians remain reluctant to de-escalate empirical therapy, fearing treatment failure. In a study of diabetic foot infections treated with surgical debridement, neither broad-spectrum therapy nor microbiologically adequate empiric therapy was shown to statistically improve clinical outcomes compared with patients initially treated with empiric inadequate antibiotic therapy [22]. Similarly, a study of heterogeneous orthopedic infections reported no increase in treatment failure among patients initially receiving empiric regimens lacking activity against subsequently identified pathogens [23].

This highlights the need to investigate whether a more tailored empirical approach across different BJI subtypes, including both antimicrobial selection and route of administration, is justified. Despite evidence that patient comorbidities influence pathogen distribution in BJIs [11,24], empiric antibiotic strategies remain largely uniform, an approach that may be overly simplistic in light of the substantial heterogeneity in host characteristics and infection biology.

Therefore, this study aimed to evaluate the outcomes and comorbidity risk factors of patients initially treated with a narrow-spectrum empirical regimen, combined with surgical debridement and local antibiotic delivery, in relation to subsequent culture-based pathogen susceptibility: an approach that, to our knowledge, has not previously been investigated.

## 2. Results

The baseline demographics between the three groups varied. Of note, 47% of the patients with culture-positive samples non-susceptible to the empiric treatment, prompting a change in antibiotic treatment (NonSus), had diabetes compared with 22% of the patients with culture-positive samples susceptible to the empiric treatment (SusEmp) and 30% of patients with culture-negative samples (CulNeg). Furthermore, 61% of NonSus patients had an ASA score of 3 or 4 compared with 42% in SusEmp and 41% in CulNeg, and 16% of NonSus had PAD compared with 7.1% in SusEmp and 7.2% in CulNeg.

Before adjustment for demographic differences (Table 1), clinical failure and proximal amputation rates differed significantly between the three groups: SusEmp (13.0%), NonSus (24.6%) and CulNeg (14.3%). The observed difference was mainly attributable to a higher cumulative incidence of proximal amputation in the NonSus group (9.3%) compared to CulNeg (3.8%) and SusEmp (1.4%). After adjustment for covariates, increasing age at the time of the primary revision remained independently associated with clinical failure, whereas diabetes was no longer statistically significant in the multivariate model (Table 2). Patients in the NonSus group had more than twice the odds of clinical failure compared to the SusEmp group (OR: 2.10; 95% CI: 1.12–3.95).

Regarding secondary proximal amputation, ASA classes 3 to 4 and PAD were independently associated with increased risk, with adjusted Odds Ratios of 4.43 (95% CI: 1.12–22.8) and 3.25 (95% CI: 1.10–10.3), respectively (Table 3). Patients in the NonSus group also demonstrated a substantially higher risk of proximal amputation compared to the SusEmp group (adjusted OR 4.95, 95% CI 1.41–23.2).

**Figure 1:**
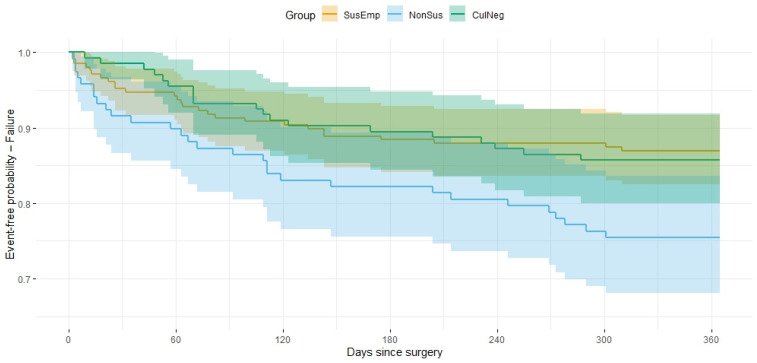
Kaplan–Meier Curve, clinical failure overall. Log-rank test (overall difference across groups): *p* = 0.018; SusEmp: susceptible patients; NonSus: non-susceptible patients; CulNeg: culture-negative patients.

**Figure 2:**
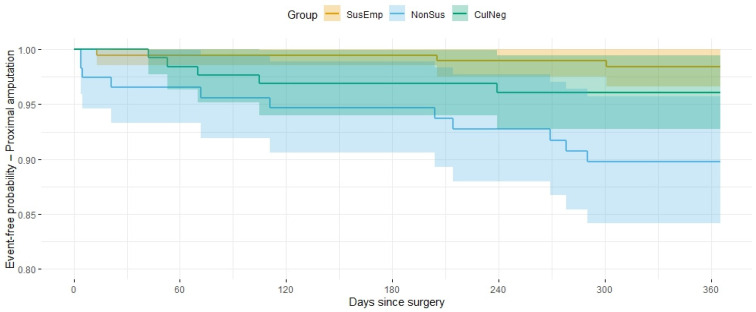
Kaplan–Meier Curve, 2nd event: proximal amputation. Log-rank test (overall difference across groups): *p* = 0.002; SusEmp: susceptible patients; NonSus: non-susceptible patients; CulNeg: culture-negative patients.

Kaplan–Meier analysis demonstrated that failure-free survival differed significantly across microbiological groups (*p* = 0.018). At 12 months, failure-free survival was 87% in the SusEmp group and 85.7% in the CulNeg group, compared with 75.4% in the NonSus group (Figure 1). Similarly, proximal amputation–free survival also differed significantly between the groups (*p* = 0.002), with 12-month amputation-free survival rates of 98.6% in SusEmp, 96.2% in CulNeg, and 90.7% in NonSus (Figure 2).

## 3. Discussion

Bone and joint infections remain one of the most challenging conditions in orthopedic surgery, often prompting the routine use of broad-spectrum empiric systemic antibiotics. This study, while employing a one-stage surgical strategy combined with high-concentration local antibiotic therapy and a narrow-spectrum Gram-positive–targeted systemic regimen, resulted in acceptable and clinically comparable outcomes for patients. Clinical failure at 12 months occurred in 13% of patients with susceptible pathogens and 14% of culture-negative cases, both well within the 10–30% failure range consistently reported in the contemporary BJI literature [18,19,20]. In contrast, patients with non-susceptible pathogens experienced worse outcomes, with a failure rate of 24.6% and nearly fivefold higher odds of proximal amputation.

In our study, we found that patients requiring changes in antibiotic therapy (NonSus) had approximately twice the odds of clinical failure compared with patients susceptible to narrow empiric antibiotics (SusEmp) (OR: 2.10). Specifically, regarding the risk of secondary amputation, we found a nearly fivefold increase in odds (OR: 4.95). These findings could indicate that as a generalized overall approach for all BJI patients, the empiric antibiotic regimen used in this study was too narrow. However, they could also potentially reflect an uneven distribution of BJI subpopulations between groups, the investigation of which unfortunately lay outside the scope of the present study.

Globally, a systemic broad-spectrum empirical antibiotic profile is generally applied in BJI cases, including coverage for Gram-negative and Gram-positive bacteria despite a predominance of Gram-positive bacteria, without differentiation regarding the comorbidities or demographics of the patients [20,25,26,27]. This broad-spectrum approach is often guided by susceptibility findings *in vitro* and overlooks the clinical importance of surgical debridement, the role of locally administered antibiotics, and the patient’s immune response [10,28,29]. It further overlooks the importance of the basic information of the patient characteristics, which our study suggests have a data-driven influence on treatment outcome. Identification of host-related factors, such as comorbidity profiles and vascular statuses, may be more predictive of treatment failure than the width of the empirical antibiotic regimen. The difference in clinical outcome shown between SusEmp and NonSus after adjusting for other factors also indicates that early microbiological mismatch is predictive of treatment failure. Furthermore, the microbiology appears to correspond closely to patient related characteristics, as reflected by the subgroup-specific distribution of comorbidities. This subgroup-dependent pattern in microbiology, reflected in the antibiotic susceptibility of the identified bacteria, not only underlines the need for clinical *in vivo* data but also indicates a more complex correlation between patient-related demographics, comorbidities, microbiology, and treatment outcomes in protocolized BJI management. These findings suggest that a one-size-fits-all model may be overly simplistic and has to be discarded.

In contrast to our findings, a recent retrospective cohort study investigating the consequences of inadequate empirical antibiotics in relation to debridement for pooled orthopedic infections did not find an increased rate of clinical failure [23]. However, they did not investigate the relationship between therapeutic failures and patient-related comorbidities.

In our study, we did not control for either the time interval from the initiation of empiric therapy to the administration of microbiologically susceptible antibiotics nor the virulence characteristics of the infecting organisms, both of which may potentially impact outcomes. Notably, Gram-negative pathogens have been associated with distinct and sometimes more aggressive osteolytic progression profiles compared to Gram-positive organisms in fracture-related infections [30]. This biological variability may partly explain the higher failure rates observed in the NonSus cohort and suggests that pathogen-specific virulence characteristics should be considered alongside antimicrobial susceptibility when interpreting treatment outcomes.

This study adds a clinically important perspective: patient-related factors (age, ASA group and PAD) are independently associated with clinical failure. Rather than applying a universal broad-spectrum approach, our data suggest that empirical treatment could potentially be stratified based on patient demographics and comorbidities. Although randomized outcome data remain limited, published retrospective cohort data support the concept that pathogen likelihood varies by host factors and clinical context [31,32,33]. This supports the feasibility of stratifying empiric therapy based on demographic and comorbidity-related risk profiles. This subgroup-driven model could enable more judicious antibiotic use with empirical treatment more suited to the patients’ likely microbiological profiles and risk of treatment failure, advancing antimicrobial stewardship without compromising clinical outcomes. In line with modern antibiotic stewardship, it is desirable to minimize liberal use of broad-spectrum systemic antibiotics, as long as the treatment provided does not result in a poorer outcome. Evidence-based use of the right antibiotic for the right patient also has a clear impact on health economics. This was demonstrated by the OVIVA trial, which showed an improvement in health economics through a reduction in hospital stays by implementing early substitution of intravenous treatment with oral antibiotics [34].

Out of all our investigated comorbidities, the only ones that were shown to significantly impact the risk of the outcome “2nd event: Proximal amputation” were ASA group and PAD. These findings are consistent with recent studies in diabetic foot populations, where PAD has been identified as a significant risk factor for clinical failure [35]. We also found a significantly higher prevalence of patients with high ASA group scores and patients diagnosed with PAD in the NonSus group, who were prompted to undergo a change in systemic antibiotics.

It is therefore relevant to investigate the relationship between comorbidities and microbiological test results, enabling treating physicians to better predict which patients would benefit from a broad-spectrum systemic antibiotic regimen. For future studies, it would be of interest to prospectively identify patients corresponding to those in our “NonSus” group based on their demographics and comorbidities and investigate whether the outcome in this patient group would improve with broad-spectrum systemic empiric antibiotic treatment. Stratification of antibiotic treatment strategies according to relevant risk factors based on comorbidity and patient demographics may therefore have the potential to reduce the unnecessary overuse of broad-spectrum systemic antibiotics without compromising the overall clinical failure rate. This supports a shift away from empirical prescribing based on interpretations of *in vitro* results toward a stratified, evidence-driven framework where empiric regimens are tailored according to patient-specific clinical data that influence the risk of failure, rather than hypothetical pathogen profiles. This represents a fundamental change in how empirical therapy in BJIs should be conceptualized.

### Limitations

This study has several inherent limitations, mainly its retrospective, single-center design, which may have limited generalizability. Patients were included with substantial heterogeneity in comorbidities and clinical BJI diagnoses (FRIs, PJIs, DFO, and cOM). We did not investigate whether the subpopulation of BJIs differentiated between our three patient groups. Time from initiation of empirical therapy to administration of a culture-directed regimen in the NonSus group was not systematically evaluated in the current study. Histopathological testing was not applied, and therefore the inflammatory aspect was not assessed. Information on antibiotic treatment prior to microbiological sampling was incomplete and not investigated further. Potential temporal changes in local antimicrobial resistance patterns during the study period were not assessed. Exclusion of patients not treated with local antibiotic bone void filler may have introduced selection bias and restricted interpretation to BJIs managed with adjunctive local antibiotic therapy. We also did not record whether the orthopedic surgeon preforming the primary operation was an infectious specialist. However, all patients with relevant BJI diagnoses who underwent surgery during the study period were included, keeping selection bias at a minimum, although not eliminating it entirely. Specific data on microbial prevalence in BJIs in Northern Europe are currently scarce, and more studies are needed to determine whether the patients in our study are representative in regard to this parameter. In the current study, we did not specifically look at microbial virulence, only susceptibility to the empiric antibiotic treatment. Specific BJI subtypes and implant retention status were not available for analysis and could therefore not be adjusted for in the multivariable models, resulting in a potential risk of residual confounding. As multiple statistical analyses were performed, the possibility of type I errors cannot be excluded, despite the use of predefined outcomes and clinically relevant covariates.

## 4. Materials and Methods

### 4.1. Cohort

All patients clinically diagnosed with BJIs (covering FRIs, cOM, PJIs, and DFO) and requiring surgery were divided into subgroups. FRIs were defined using the international FRI consensus definition [36], cOM was defined as infection for more than 6 weeks with a sinus tract or draining wound in relation to dead bone and inflammatory features [37], PJIs were defined according to the European Bone and Joint Infection Society (EBJIS) criteria [5], and DFO was diagnosed based on a positive probe-to-bone test in combination with radiographic findings, with magnetic resonance imaging used when required [35]. All patients were surgically treated at a specialized orthopedic infection unit between February 2016 and April 2023 (Figure 1). This study was conducted as a retrospective cohort study comparing clinical outcomes and demographics in three patient groups: “SusEmp”, patients with culture-positive samples susceptible to the empiric treatment; “NonSus”, patients with culture-positive samples non-susceptible to the empiric treatment, prompting a change in antibiotic treatment; and “CulNeg”, patients with culture-negative samples while still receiving empirical antibiotics. This study was conducted in accordance with Danish regulations. As such, approval from the regional research ethics committee was not required. The study protocol was reviewed and approved by the hospital administration and the institutional legal department to ensure compliance with data protection regulations.

**Figure 3:**
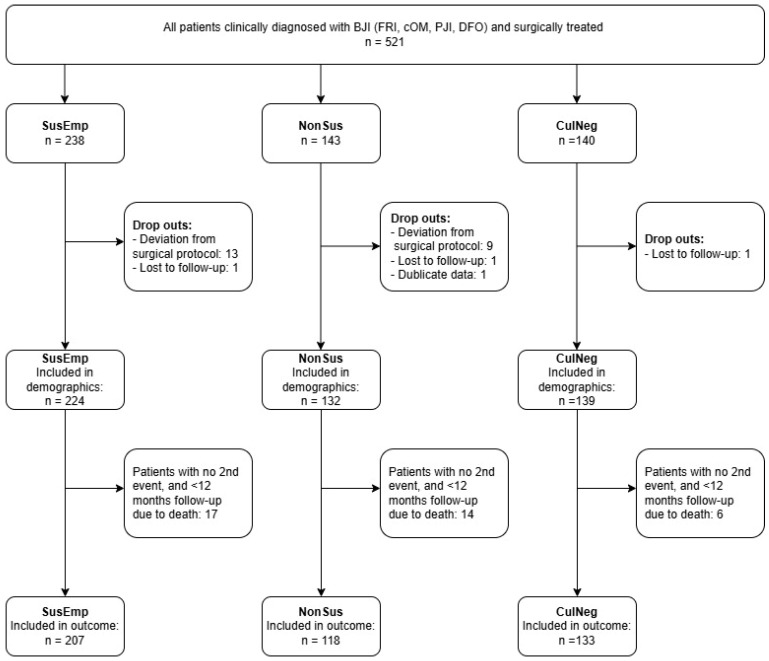
Flowchart diagram of the study cohort. Illustrates patient screening, inclusion and allocation. BJI: bone and joint infection, FRI: fracture-related infection, cOM: chronic osteomyelitis, PJI: prosthetic joint infection, DFO: infected diabetic foot osteomyelitis, SusEmp: susceptible patients, NonSus: non-susceptible patients, CulNeg: culture-negative patients.

Patients were categorized based on sex, age at primary operation (OP age); Body Mass Index (BMI); American Society of Anesthesiologists (ASA) group; active smoking; reported alcohol abuse; reported drug abuse; and comorbidity status regarding diabetes, cardiovascular disease, peripheral arterial disease (PAD), endocrinological disease (other than diabetes), Central Nervous System (CNS) disease, psychological diagnoses, active cancer, active and/or recent immunocompromising treatment, pulmonary disease, renal disease, and gastrointestinal disease (Table 1). Patients who did not receive local bone void filler with antibiotics during primary revision surgery were excluded (n = 22). Three (0.58%) patients were excluded due to loss to follow-up, and one (0.19%) entry was excluded from the database due to “Duplicate entry” (Figure 3).

### 4.2. Surgical Procedure

In brief, the surgical model was modeled from a one-stage protocol developed at Oxford’s Bone Infection Unit [38]. Surgical management of DFO followed the established one-stage surgery principles of BJI treatment, the CLOSE-UP procedure [35]. Surgical management involved excision of necrotic skin and previous scars, followed by exposure of infected foci. A minimum of five deep tissue samples (bone and soft tissue) were collected from the infected area using sterile instruments, with a separate instrument for each sample to prevent cross-contamination [39]. No histological samples were taken, as this is not part of routine diagnostic practice in Denmark. Surgical debridement was performed until viable, bleeding, well-perfused, and mechanically stable bone was observed [40]. When clinically indicated in DFO, amputation or joint exarticulation was performed to achieve infection control and preserve function. Following debridement, the surgical site was irrigated with copious amounts of sterile sodium chloride solution. Local antibiotic therapy was administered in the form of a calcium sulfate/hydroxyapatite composite impregnated with gentamicin or vancomycin (CeramentG: 17.5 mg gentamicin/mL; CeramentV: 66 mg vancomycin/mL; Bonesupport AB, Lund, Sweden). Wounds were primarily closed where feasible, ensuring tension-free closure. When necessary, the surgery was conducted in collaboration with a plastic surgeon.

### 4.3. Systemic Antibiotic Strategy

Following tissue sampling, all patients received empiric intravenous (IV) antibiotic therapy—penicillin G (2 million IU) and cloxacillin (1000 mg), each administered four times daily. In cases of penicillin allergy, patients received intravenous cefuroxime (750 mg, four times daily). The narrow-spectrum Gram-positive empirical regimen was prescribed according to regional recommendations and defined in collaboration with local microbiologists and infectious disease specialists. The duration of initial intravenous therapy was seven days for all patients, followed by a switch to oral antibiotics, according to the OVIVA study protocol [41].

If the isolated pathogen was not susceptible to the empirical regimen, for example, Gram-negative organisms or *Staphylococcus epidermidis*, antimicrobial therapy was modified accordingly. The change occurred immediately after notification of susceptibility results from the microbiology laboratory. Patients then received seven days of appropriate intravenous treatment before transitioning to a targeted oral regimen determined by an infectious disease specialist. For polymicrobial cultures, patients were classified into the NonSus group if at least one isolated organism was not susceptible to the empirical regimen. If all isolated pathogens were susceptible, patients were assigned to the SusEmp group. In cases of mixed susceptibility, the most resistant pathogen determined group assignment. Oral antibiotic therapy was subsequently continued for either five or eleven weeks, depending on whether the metalwork was removed or retained, with the longer duration applied in cases of implant retention.

### 4.4. Outcome

Clinical failure was defined as the need for revision surgery or amputation based on clinical signs of an ongoing infection within 12 months of the initial intervention. These included a persistent non-healing wound, draining sinus tract, purulence, non-union, prosthesis or implant loosening, or fracture failure [13,41].

Secondary outcomes were subcategorized as “surgical revision”, defined as debridement of bone or soft tissue, or “proximal amputation”, defined as an amputation more proximal than the primary revision surgery, that being a below- or above-knee amputation.

### 4.5. Statistics

Baseline characteristics were summarized using the median (Q1–Q3) for continuous variables and n (%) for categorical variables. Categorical group comparisons were performed using the chi-squared test. If any expected cell count was below five, Fisher’s exact test was applied instead. Continuous variables were assessed for normality using the Shapiro–Wilk test and for homogeneity of variances using Levene’s test. Due to the skewed distributions of BMI and OP age, the Kruskal–Wallis test was used, with data reported as medians (Q1–Q3).

Potential confounders were assessed using univariable logistic regression, and variables with *p*-values < 0.05 were subsequently included in the multivariable logistic regression model, adjusting for demographic factors that showed statistically significant differences between groups. This approach allowed estimation of the independent effect of each variable while adjusting for other covariates, thereby identifying true predictors of outcome rather than associations driven by correlated risk factors. This was done for each outcome separately. The SusEmp group was used as the reference against NonSus and CulNeg to calculate adjusted Odds Ratios (aORs). All multivariable models demonstrated acceptable calibration according to the Hosmer–Lemeshow test (all *p* > 0.05).

Only patients who experienced clinical failure or had a minimum of 12 months of follow-up were included in the outcome analyses and logistic regression models. Patients who experienced clinical failure before death were counted as failures. In cases where data on patient characteristics were missing, N was reduced to only include the patients for whom data were available. All analyses were conducted in R version [4.2.3]. *p* values < 0.05 were considered statistically significant.

## 5. Conclusions

We have demonstrated that a large group of patients can presumably be treated safely with a more restrictive narrow-spectrum approach, based on the clinical outcomes of our surgical and medical procedures. We also found that there is a group of high-risk patients who could potentially be identified based on demographics and comorbidities, for whom a broad-spectrum antibiotic treatment may be relevant. More studies are needed to identify the subgroups suited for safe use of a narrow-spectrum empiric regimen, thereby reserving broad-spectrum antibiotics for patients with the appropriate indications and for whom they would have a positive effect on clinical outcome. This approach would justify more restrictive stewardship of broad-spectrum antibiotic use without negatively impacting patient outcome.

## Figures and Tables

**Table 1:** Demographics.

Characteristic ^1^	SusEmp N ^3^ = 224	NonSus N ^3^ = 132	CulNeg N ^3^ = 139	*p*-Value
Sex, male	134 (60%)	88 (67%)	69 (50%)	**0.016**
OP age	66 (50–76)	66 (58–77)	63 (49–73)	**0.035**
BMI	25.0 (22.0–28.0)	26.0 (23.0–29.0)	26.0 (23.0–31.0)	**0.020**
ASA group 3–4	94 (42%)	81 (61%)	57 (41%)	**<0.001**
Active smoking	49 (23%)	33 (25%)	28 (21%)	0.9
Reported alcohol abuse	26 (12%)	17 (13%)	15 (11%)	0.076
Reported drug abuse	6 (2.7%)	3 (2.3%)	1 (0.7%)	0.4
Diabetes	50 (22%)	62 (47%)	42 (30%)	**<0.001**
Cardiovascular disease	110 (49%)	80 (61%)	67 (48%)	0.065
Peripheral arterial disease	16 (7.1%)	21 (16%)	10 (7.2%)	**0.013**
Other endo. disease	50 (22%)	46 (35%)	43 (31%)	**0.027**
CNS disease	25 (11%)	23 (17%)	14 (10%)	0.13
Psychological diagnoses	20 (8.9%)	18 (14%)	9 (6.5%)	0.12
Active cancer	14 (6.3%)	10 (7.6%)	4 (2.9%)	0.3
Immun. supp. treatment	22 (9.8%)	8 (6.1%)	6 (4.3%)	0.13
Pulmonal disease	22 (9.8%)	14 (11%)	6 (4.3%)	0.093
Renal disease	15 (6.7%)	10 (7.6%)	9 (6.5%)	>0.9
Gastrointestinal disease	13 (5.8%)	9 (6.8%)	4 (2.9%)	0.3
**Primary outcome** ^1,2^				
Clinical failure	27 (13.0%)	29 (24.6%)	19 (14.3%)	**0.019**
**Secondary outcome** ^1,2^				
2nd event: Surgical revision	24 (11.6%)	18 (15.3%)	14 (10.5%)	0.49
2nd event: Proximal amputation	3 (1.4%)	11 (9.3%)	5 (3.8%)	**0.004**

^1^ Categorical variable: chi-squared test of independence, n/N (%); if expected cell count was <5, Fisher’s exact test, n/N (%), was applied. Continuous variables: Kruskal–Wallis test, median (Interquartile Range (Q1–Q3)). ^2^ SusEmp: N = 207; NonSus: N = 118; CulNeg: N = 133. ^3^ If missing patient data, N was adjusted to the number of patients with data collected. Bold face: *p* < 0.05. Abbreviations: OP age—age at primary operation, BMI—Body Mass Index, ASA—American Society of Anesthesiologists, PAD—peripheral arterial disease, Other endo. disease—endocrinological disease (other than diabetes), CNS—Central Nervous System, Immun. supp. treatment—recent immunocompromising treatment, SusEmp—susceptible patients, NonSus—non-susceptible patients, CulNeg—culture-negative patients.

**Table 2:** Factors associated with clinical failure: unadjusted and adjusted Odds Ratios.

Characteristic ^1^	Univariate OR			Multivariate aOR		
	OR	95% CI	*p*-Value	aOR	95% CI	*p*-Value
Sex, male	1.30	0.79, 2.20	0.31	1.05	0.61, 1.83	0.87
OP age	0.99	0.98, 1.00	0.15	0.98	0.97, 1.00	**0.04**
BMI	1.03	0.98, 1.07	0.21	1.02	0.98, 1.07	0.316
ASA group 3–4	1.28	0.78, 2.11	0.33	1.09	0.58, 2.05	0.79
Diabetes	1.82	1.08, 3.02	**0.022**	1.66	0.89, 3.07	0.11
Peripheral arterial disease	1.45	0.63, 3.05	0.36	1.39	0.55, 3.32	0.47
Other endo. disease	1.02	0.57, 1.75	0.96	0.98	0.51, 1.83	0.96
**Group factor:**						
NonSus vs. SusEmp	2.17	1.21, 3.91	**0.009**	2.10	1.12, 3.95	**0.02**
CulNeg vs. SusEmp	1.11	0.58, 2.08	0.74	1.13	0.58, 2.18	0.71

^1^ If missing patient data, N was adjusted to the number of patients with data collected. Bold face: *p* < 0.05. Abbreviations: OR—Odds Ratio, aOR—adjusted Odds Ratio, CI—Confidence Interval, OP age—age at primary operation, BMI—Body Mass Index, ASA—American Society of Anesthesiologists, PAD—peripheral arterial disease, Other endo. disease—endocrinological disease (other than diabetes), CNS—Central Nervous System, Immun. supp. treatment—recent immunocompromising treatment, SusEmp—susceptible patients, NonSus—non-susceptible patients, CulNeg—culture-negative patients.

**Table 3:** Risk factors for secondary proximal amputation: unadjusted and adjusted Odds Ratios.

Characteristic ^1^	Univariate OR			Multivariate aOR		
	OR	95% CI	*p*-Value	aOR	95% CI	*p*-Value
Sex, male	1.55	0.60, 4.48	0.38	0.99	0.35, 3.04	0.98
OP age	1.02	0.99, 1.05	0.19	1.00	0.96, 1.04	0.86
BMI	1.01	0.93, 1.09	0.82	1.00	0.91, 1.09	0.99
ASA group 3–4	7.23	2.37, 31.4	**0.002**	4.43	1.12, 22.8	**0.047**
Diabetes	3.38	1.34, 8.91	**0.011**	1.32	0.45, 3.97	0.61
Peripheral arterial disease	6.73	2.38, 17.9	**<0.001**	3.25	1.10, 10.3	**0.043**
Other endo. disease	1.30	0.45, 3.37	0.60	0.64	0.20, 1.89	0.43
**Group factor:**						
NonSus vs. SusEmp	6.99	2.13, 31.4	**0.003**	4.95	1.41, 23.2	**0.021**
CulNeg vs. SusEmp	2.66	0.64, 13.1	0.19	2.67	0.60, 13.9	0.20

^1^ If missing patient data, N was adjusted to the number of patients with data collected. Bold face: *p* < 0.05. Abbreviations: OR—Odds Ratio, aOR—adjusted Odds Ratio, CI—Confidence Interval, OP age—age at primary operation, BMI—Body Mass Index, ASA—American Society of Anesthesiologists, PAD—peripheral arterial disease, Other endo. disease—endocrinological disease (other than diabetes), CNS—Central Nervous System, Immun. supp. treatment—recent immunocompromising treatment, SusEmp—susceptible patients, NonSus—non-susceptible patients, CulNeg—culture-negative patients.

## Data Availability

Due to privacy concerns regarding protected health information, the data presented in this study are not publicly available. Availability can be obtained upon request from the corresponding author. Statistical analyses were performed using R, version [4.2.3], and the code is available upon request.

## Abbreviations

The following abbreviations are used in this manuscript:

AMR	Antimicrobial resistance
aOR	Adjusted Odds Ratio
ASA	American Society of Anesthesiologists
BJI	Bone and joint infection
BMI	Body Mass Index
CI	Confidence Interval
CNS	Central Nervous System
cOM	Chronic osteomyelitis
CulNeg	Patients with culture-negative samples
DFO	Diabetic foot osteomyelitis
EBJIS	European Bone and Joint Infection Society
FRI	Fracture-related infection
Immun. supp. treatment	Recent immunocompromising treatment
IU	International Unit
IV	Intravenous
mg	Milligram
NonSus	Patients with culture-positive samples non-susceptible to the empiric treatment, prompting a change in antibiotic treatment
OP age	Age at primary operation
OR	Odds Ratio
“Other endo. Disease”	Endocrinological disease (other than diabetes)
OVIVA	Oral Versus Intravenous Antibiotics for Bone and Joint Infection trial
PAD	Peripheral arterial disease status
PJI	Prosthetic joint infection
SusEmp	Patients with culture-positive samples susceptible to the empiric treatment
UI	Uncertainty interval
